# Use of item response theory to develop a shortened version of the EORTC QLQ-BR23 scales

**DOI:** 10.1038/s41598-018-37965-x

**Published:** 2019-02-11

**Authors:** Juan Xia, Zheng Tang, Peng Wu, Jiwei Wang, Jinming Yu

**Affiliations:** 1 Institute of Clinical Epidemiology, Key Laboratory of Public Health Safety, Ministry of Education, School of Public Health, Fudan University, 130# DongAn Road, Shanghai, 200032 China; 2 Key Lab of Health Technology Assessment of Ministry of Health, School of Public Health, Fudan University, 130 Dong-An Road, 200032 Shanghai, China

## Abstract

It is important that questionnaires are as short as possible while still capturing the scope of problems relevant in an effective and reliable manner, to minimize the response burden. The purpose of our study was to develop a shortened version of the EORTC QLQ-BR23 for using in breast cancer survivors. Our data come from 10794 breast cancer survivors who completed the EORTC QLQ-BR23. Two-thirds of the sample was randomly selected from the original sample for development, and the remaining was used for validation. Item response theory methods were applied to shorten scales. The graded response model of Samejima was used to fit the item responses. The shortened scale was evaluated with the validation set by examining the mean difference, the proportion of respondents correctly predicted, correlation and weighted kappa between the shortened form and the original observed scores. Results reveal that a three-item BRBI, a four-item BRST, a three-item BRBS and a two-item BRAS forecast the scores on the original scales with wonderful consistency and are alike in measurement precision with no loss or only little loss in detecting group differences. Prospective validation on new diagnosed breast cancer patients and with poor QOL is needed.

## Introduction

The development of item response theory (IRT) has reached a point where testing applications^1,2^, whether in educational^3–5^ or psychological^6–9^ testing programs or in research, can be performed entirely with IRT methods. Nevertheless, IRT has only come into application a short while ago in the field of health outcomes instruments^10–14^. According to previous researches, IRT methods have obvious advantages compared with classical test theory^2,15,16^. A crucial distinction between IRT and classical test theory is that IRT defines a scale for the potential variable being measured by a set of items, and items are calibrated as for the same scale. Therefore, using IRT method can easily calibrate two assessments of different lengths^17,18^.

The European Organization for Research and Treatment of Cancer (EORTC) Breast Cancer-Specific Quality of Life Questionnaire (QLQ-BR23) is one of the most widely used supplementary questionnaire modules for evaluating the quality of life in breast cancer patients in particular^19^. The EORTC QLQ-BR23 consists of 23 items. In most cases, breast cancer patients are usually extremely ill and too weak to complete the entire wordy questionnaire in a given short time. Therefore, the brevity of the questionnaires, following with non-inferior validity and reliability, is of great importance for researchers to lower the response burden they might encountered. The goal of this study was to evaluate the possibilities for shortening the EORTC QLQ-BR23 (body image, systemic therapy side effects, breast symptoms, arm symptoms) scales for using in breast cancer survivors while still be able to compare the results of the shortened scales with the non-shortened scales firsthand.

## Materials and Methods

### Study design and sample

The example data come from 10794 breast cancer survivors from a cross-sectional study conducted in 2013, who were the member of the affiliated groups of Cancer Recovery Clubs in 34 cities across China. Informed written consent was obtained before we start the investigation from each participant. Approval for the study was received from the Ethic Committee of Public Health School of Fudan University (protocol number RB # 2013-04-0450). More detailed information on this study were available in the previous paper^20^. The sample was split into two types: one for development (2/3 of the entire sample) and the other for validation (1/3 of the entire sample).

### Questionnaire

The EORTC QLQ-BR23 consists of 23 items^21^. Twenty of the items constitute five scales and three single-items symptom measures. The sexual function only have two items and the estimation procedure could not converge, therefore, our study here is on the body image (BRBI), systemic therapy side effects (BRST), breast symptoms (BRBS) and arm symptoms (BRAS) scales. These consist of four, seven, four and four items, respectively. Each item has four response categories: “Not at All” = 1, “A Little” = 2, “Quit a Bit” = 3, and “Very Much” = 4. The scale scores are constructed by averaging items within scales and transforming average scores linearly, ranging from 0 to 100. The procedure is as follows: 1) Raw score: estimate the average of the item that contribute to the scale; 2) Linear transformation: use a linear transformation to standardize the raw score, so that scores range from 0 to 100 (functional scales: S = {1 − (Raw score − 1)/Range} * 100, symptom scales/items: S = {(Raw score − 1)/range} * 100, Range is the difference between the maximum possible value of RS and the minimum possible value). For the missing value, if less than half of the items from the scale have been answered, we set scale score to missing; if no, we using the mean value of the answered items to replace the missing items. And for single-item measures, set missing value to missing^22^.

### Statistical methods

IRT-based methods were used to shorten scales. As the response of the items are polytomous and ordered, with scoring categories ranging from one to four, we used the gradual response model of Samejima (GRM)^23^ to fit the item responses. One of the most important assumptions of the application of IRT analysis is unidimensional. We used the factor analysis to test the unidimensionality of the EORTC QLQ-BR23 scales. The results show that the scales are sufficiently unidimensional for application of unidimensional IRT analysis.

Item parameter estimates were carried out using STATA software program with the marginal maximum likelihood method. This method supposes that, for a given item n, the probability of choosing a category m or higher (with m = 2,3, …, k_n_) is specified as a logistic function of theta (θ) as

$${\rm{P}}({{\rm{x}}}_{{\rm{in}}}\ge {\rm{m}}|{\rm{\theta }},\,{{\rm{a}}}_{{\rm{n}}},{{\rm{b}}}_{{\rm{nm}}})=1/(1+\exp (-{\rm{D}}\,{{\rm{a}}}_{{\rm{n}}}({\rm{\theta }}-{{\rm{b}}}_{{\rm{nm}}})))$$

where θ represents the potential ability of the individual, an individual who have a better QOL would have a higher θ score, namely the latent level of quality of life; a_n_ is the slope parameter, represents the discrimination of the item; b_nm_ is the category threshold parameter, represents the difficulty of the item, can be interpreted as the θ value at which exactly 50 percent of the population scores in category m or higher; D is the scale constant specifying the metric of the potential disability scale, and in the conventional logistic metric D is equal to 1.7. Samejima (1969) further defines P (x_in_ ≥ 1) = 1 and P (x_in_ ≥ k_n_ + 1) = 0, therefore, the probability of observing a specific category m for a given disability θ is then equal to

$${P(x}_{{\rm{in}}}={\rm{m}}|{\rm{\theta }})={\rm{P}}\,({{\rm{x}}}_{{\rm{in}}}\ge {\rm{m}}|{\rm{\theta }})\mbox{--}{\rm{P}}\,({{\rm{x}}}_{{\rm{in}}}\ge {\rm{m}}+1|{\rm{\theta }})$$

for all m = 1, 2, …, k_n_. The item information functions (IIFs) is a measure of how much information an item provides about the IRT score. More details about the explanations of parameter refer to previous research^23^. The IIFs and the ability to predict scores on the full scales were used to select the items for the shortened scales. Item Characteristic Curves are the trace lines for each response choice, which plot how the individual items function in relation to the quality of life (the underlying trait). Difficulty and discrimination are two properties of the item characteristic curves. The parameter of difficulty describes where the item functions along the ability scale; and the parameter of discrimination of the item describes how well an item can differentiate between individuals having abilities above the item location and those having abilities below. Both the parameter of slope and the location of the items were considered during item removing.

Items were examined by subscale to determine which items to remove in the development of a shortened version of the EORTC QOL-BR23. We compared the shortened scales scores with the full scales scores by calculating the difference in mean scores; the percentage of correctly predicted groups; the Pearson correlation r, and the weighted k measure of agreement between the shortened and full scale scores.

### Ethical approval

All procedures performed in studies involving human participants were in accordance with the ethical standards of Public Health School of Fudan Univeristy (protocol number RB # 2013-04-0450) and with the 1964 Helsinki declaration and its later amendments or comparable ethical standards.

### Informed consent

Informed consent was obtained from all individual participants included in the study.

## Results

### Demographic and clinical characteristics

Of the 10794 participants in the database, two-thirds of the sample (7196) was randomly selected from the total sample for simulation, and the remaining one-third (3598) is used for verification. The sample characteristics were reported in Table 1. Approximately 90 percent of the participants aged from 50 to 70. With the TNM system used for the evaluation of the stage of disease, T represents the size of the original (primary) tumor and whether it has invaded nearby tissue; N represents nearby (regional) lymph nodes that are involved; M represents distant metastasis (spread of cancer from one part of the body to another). We found that more than 70 percent of the participants were in an early stage of the disease (TNM classification 0 or 1 or 2) and 23% (development set) and 25% (validation set) were in stage 3 or 4. The most prevalent primary treatment was surgery combined with chemotherapy, followed by surgery combined with chemotherapy and radiotherapy. Slightly more than half of the breast cancer survivors survived more than 5 years, and 22% survived over 10 years.

**Table 1:** Clinical characteristics of the 10794 subjects N (%).

	Training set (N = 7196)	Testing set (N = 3598)
Age		
Below 40 years	85 (1.18%)	46 (1.28%)
40–49 years	496 (6.89%)	222 (6.17%)
50–59 years	5615 (78.03%)	2852 (79.27%)
60–69 years	830 (11.53%)	412 (11.45%)
Above 69 years	170 (2.36%)	66 (1.83%)
Stage		
TNM Stage0	3460 (48.08%)	1655 (46.00%)
TNM Stage1	1464 (20.34%)	735 (20.43%)
TNM Stage2	628 (8.73%)	306 (8.50%)
TNM Stage3	1398 (19.43%)	755 (20.98%)
TNM Stage4	246 (3.42%)	147 (4.09%)
Treatment		
Surgery	419 (5.82%)	215 (5.98%)
Chemotherapy	228 (3.17%)	106 (2.95%)
Radiotherapy	41 (0.57%)	19 (0.53%)
Surgery + Chemotherapy	3356 (46.64%)	1736 (48.25%)
Surgery + Radiotherapy	178 (2.47%)	74 (2.06%)
Chemotherapy + Radiotherapy	194 (2.70%)	81 (2.25%)
Surgery + Chemotherapy + Radiotherapy	2672 (37.13%)	1328 (36.91%)
Other treatment	108 (1.50%)	39 (1.08%)
Time after diagnosis (years)		
0–1	682 (9.48%)	347 (9.64%)
2–5	2596 (36.08%)	1277 (35.49%)
6–10	2294 (31.88%)	1169 (32.49%)
11–	1624 (22.57%)	805 (22.37%)

### Item content and information by item

The number of the non-missing responses, mean scores, standard deviations (SD) and the information by item for the EORTC QOL-BR23 items were listed in Table 2. The item scores were transformed to a 0–100 scale, the mean scores ranged from 63.26 to 90.62, with SD ranging from 17.20 to 31.92. The information of each item within the range of −2 to 2 was shown as Table 2. Among the 18 items for the IRT analysis, the mean information of body image ranged from 0.69 to 1.47. All of the 7 items in the Systemic therapy side effects had a lower information, ranging from 0.24 to 0.55. Only one item of breast symptoms had a lower information (0.46), and the other three items all had a higher information ranging from 0.90 to 1.12. For the arm symptoms scale, the mean information ranged from 0.67 to 0.91.

**Table 2:** Item wording, subject numbers, non-missing responses, and mean scores.

Variable	Non-missing. %	Mean score	SD	θ					Mean Info.
				−2.0	−1.0	0	1.0	2.0	
**Functional scale–Body image**									
I9: Have you felt physically less attractive as a result of your disease or treatment	7055 (98.04%)	73.07	24.43	0.15	0.86	1.04	1.09	1.36	0.69
I10: Have you been feeling less feminine as a result of your disease or treatment?	7057 (98.07%)	68.46	28.18	0.05	1.48	1.93	3.34	2.39	1.19
I11: Did you find it difficult to look at yourself naked?	7069 (98.24%)	63.26	31.92	0.03	2.15	2.14	5.26	0.59	1.47
I12: Have you been dissatisfied with your body?	7059 (98.10%)	66.32	29.98	0.05	1.75	1.86	3.88	1.32	1.20
**Symptom scales**									
**Systemic therapy side effects**									
I1: Did you have a dry mouth?	7108 (98.78%)	77.85	21.12	0.16	0.39	0.46	0.35	0.46	0.34
I2: Did food and drink taste different than usual?	7047 (97.93%)	90.62	17.20	0.02	0.10	0.35	0.60	0.54	0.33
I3: Were your eyes painful, irritated or watery?	7084 (98.44%)	81.37	21.68	0.09	0.31	0.56	0.47	0.55	0.37
I4: Have you lost any hair?	7069 (98.24%)	80.99	23.28	0.10	0.22	0.34	0.33	0.35	0.24
I6: Did you feel ill or unwell?	7028 (97.67%)	80.99	21.58	0.07	0.40	0.93	0.68	0.99	0.55
I7: Did you have hot flushes?	7051 (97.98%)	88.86	18.37	0.04	0.13	0.36	0.49	0.44	0.28
I8: Did you have headaches?	7050 (97.97%)	84.43	19.73	0.07	0.25	0.55	0.51	0.51	0.36
**Breast symptoms**									
I20: Have you had any pain in the area of your affected breast?	7054 (98.03%)	79.54	21.35	0.04	0.56	1.88	0.86	2.11	0.90
I21: Was the area of your affected breast swollen?	7000 (97.28%)	85.96	20.41	0.00	0.11	2.04	1.41	3.07	1.12
I22: Was the area of your affected breast oversensitive?	6985 (97.07%)	84.18	20.57	0.01	0.23	1.79	1.06	2.10	0.91
I23: Have you had skin problems on or in the area of your affected breast (e.g., itchy, dry, flaky)?	7010 (97.42%)	82.56	21.71	0.07	0.31	0.73	0.63	0.78	0.46
**Arm symptoms**									
I17: Did you have any pain in your arm or shoulder?	7067 (98.21%)	73.31	23.91	0.10	1.14	1.40	1.54	2.11	0.91
I18: Did you have a swollen arm or hand?	7064 (98.17%)	77.58	25.14	0.05	0.53	1.61	1.31	1.85	0.77
I19: Was it difficult to raise your arm or to move it sideways?	7058 (98.08%)	81.57	23.03	0.04	0.34	1.28	1.00	1.47	0.67

**Figure 1:**
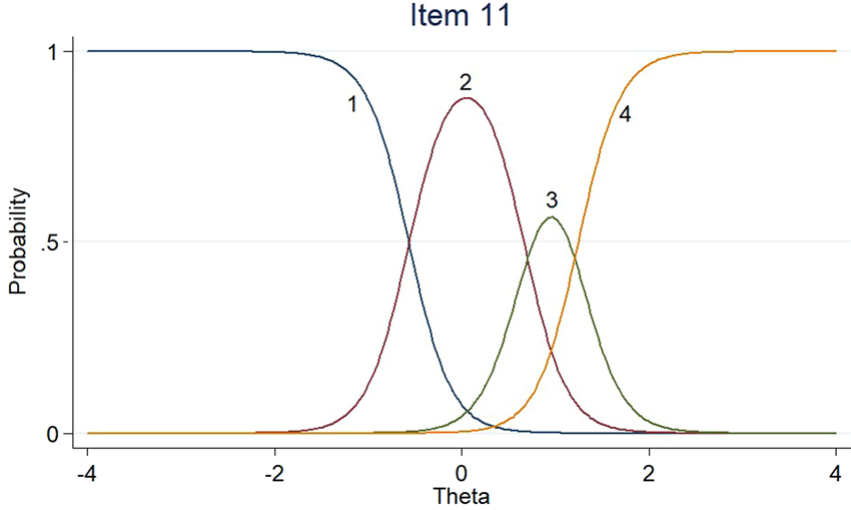
Item characteristic curves – Item 11.

**Figure 2:**
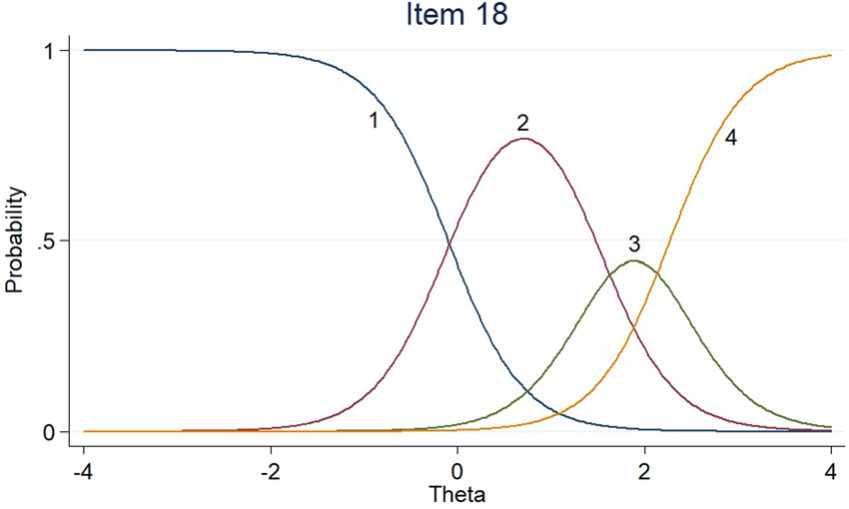
Item characteristic curves – Item 18.

**Figure 3:**
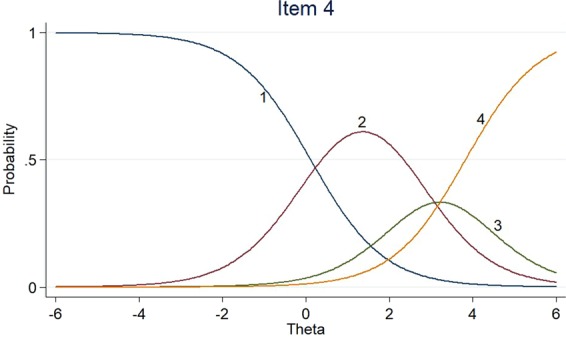
Item characteristic curves – Item 4.

Figures 1–3 listed the category characteristic curves (CCCs), showing how items relate to the ability, for three of the 18 EORTC QLQ-BR23 scale items. These items were selected to show how CCCs varied depending on the slope parameter. Item 11 had a higher slope (a = 4.44), the slope of item 18 was moderate (a = 2.53), and the slope of item 4 was low (a = 1.14). For the location parameter, the response categories for items 4 and 18 were endorsed at higher levels of QOL. The CCCs of other items could be found in the Supplementary Material.

**Figure 4:**
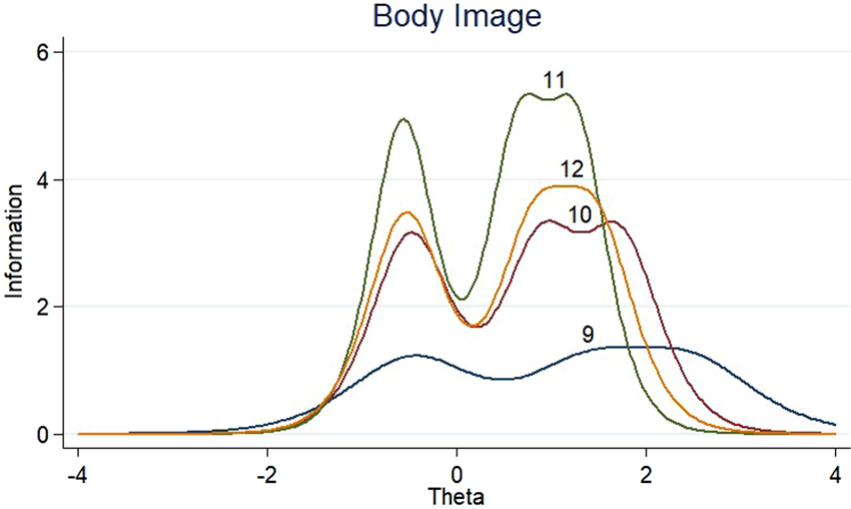
Item information functions for BRBI.

**Figure 5:**
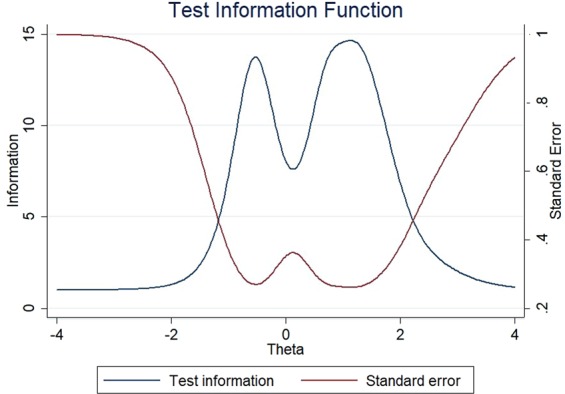
Test information functions for BRBI.

**Figure 6:**
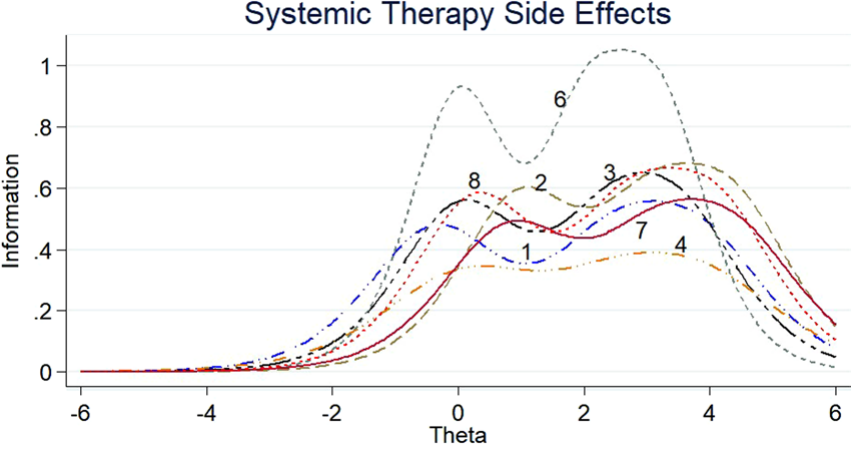
Item information functions for BRST.

**Figure 7:**
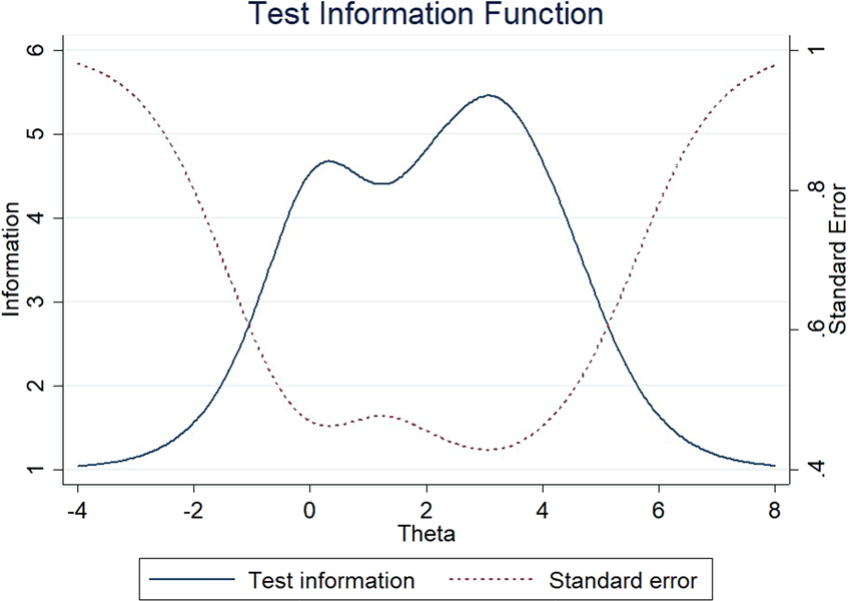
Test information functions for BRST.

**Figure 8:**
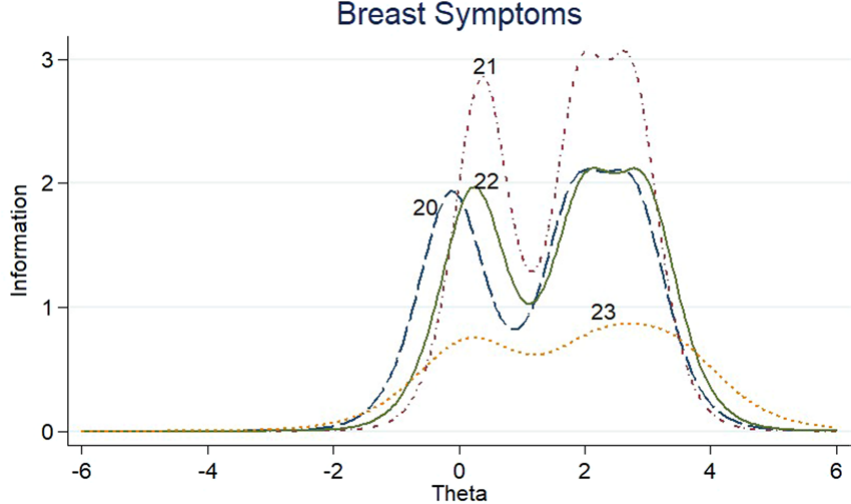
Item information functions for BRBS.

**Figure 9:**
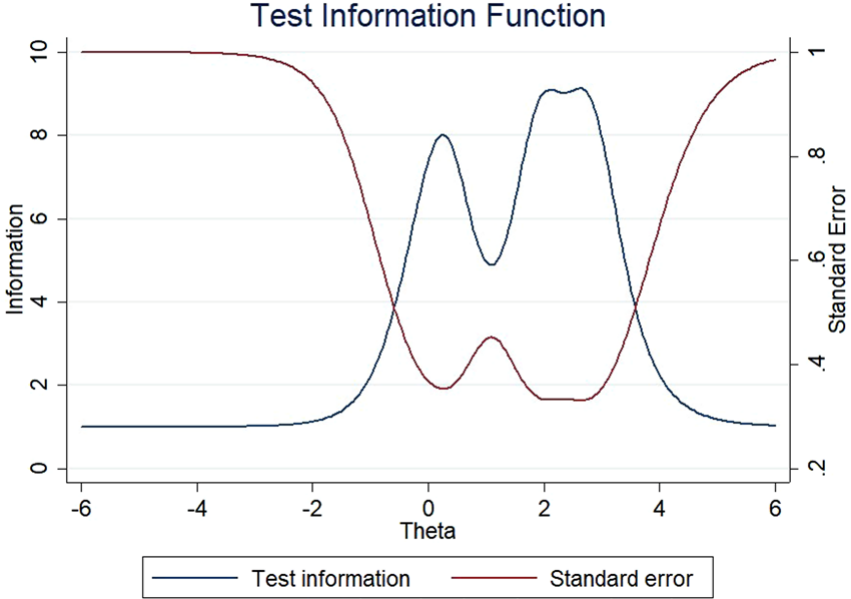
Test information functions for BRBS.

**Figure 10:**
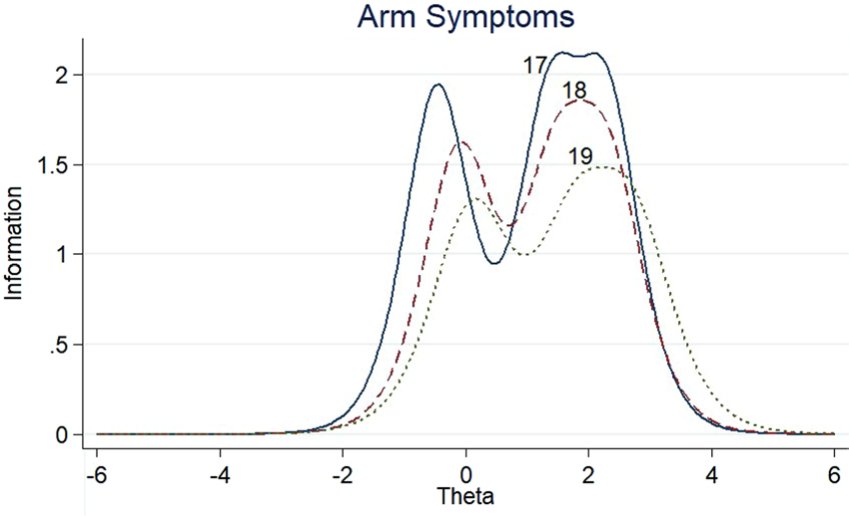
Item information functions for BRAS.

**Figure 11:**
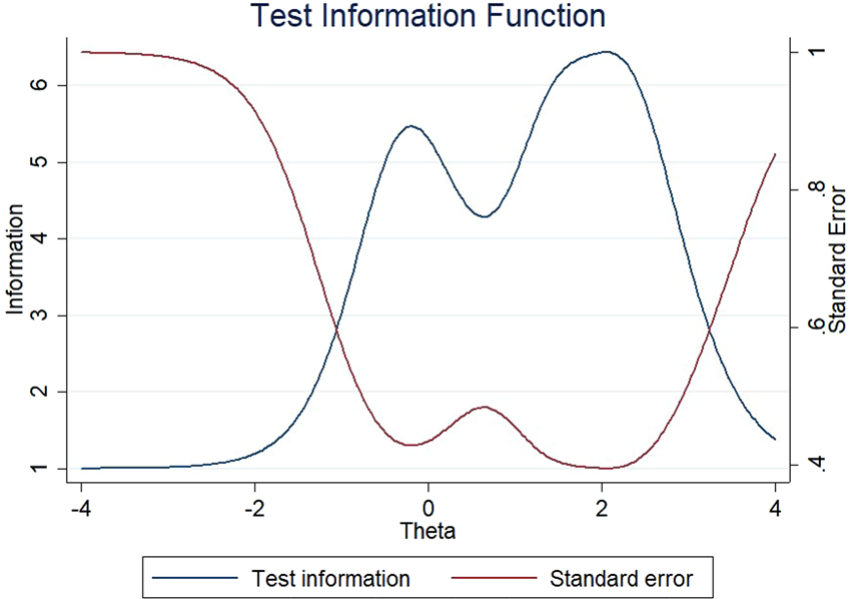
Test information functions for BRAS.

The item information functions (IIFs) and test information function (TIF) were displayed in Figs 4–11. IIFs demonstrated the precision and information that was provided by each item. TIF provided the summation of the item level information. Items with the most information for each scale were selected. For the body image scale which had the largest amount of information, we retained item 11, 12 and 10; for systemic therapy side effects scale, we selected item 6, 8, 2 and 3; for breast symptoms scale, we retained item 21, 20 and 22; for arm symptoms, we selected item 17 and 18.

### Item properties

The estimation of item parameters from the GRM calibration were showed for each item in Table 3. The estimation of slope ranged from 1.14 to 4.44, showing a great variability in discrimination among all the items. The threshold estimates for each item were presented in an increasing order, and there were no inverse threshold values. The threshold estimates endorsing 1 versus ≥2 (b_1_) ranged from −0.56 to 0.99, and endorsing 2 versus ≥3 (b_2_) ranged from 0.67 to 3.18, and endorsing 3 versus 4 (b_3_) ranged from 1.25 to 4.32.

**Table 3:** Graded response model item parameters (Coefficient (Standard Error)).

	a	b1	b2	b3
**Body image**				
I 9	2.20 (0.06)	−0.46 (0.02)	1.43 (0.03)	2.44 (0.05)
I 10	3.55 (0.10)	−0.48 (0.02)	0.91 (0.02)	1.71 (0.03)
I 11	4.44 (0.15)	−0.56 (0.02)	0.67 (0.02)	1.25 (0.02)
I 12	3.72 (0.10)	−0.53 (0.02)	0.85 (0.02)	1.45 (0.03)
**Systemic therapy side effects**				
I 1	1.37 (0.04)	−0.36 (0.02)	2.53 (0.07)	3.74 (0.12)
I 2	1.52 (0.06)	0.99 (0.03)	3.11 (0.09)	4.29 (0.17)
I 3	1.48 (0.05)	0.05 (0.02)	2.48 (0.07)	3.45 (0.10)
I 4	1.14 (0.04)	0.12 (0.03)	2.60 (0.08)	3.82 (0.13)
I 6	1.91 (0.06)	0.02 (0.02)	2.10 (0.05)	3.16 (0.09)
I 7	1.38 (0.05)	0.82 (0.03)	3.18 (0.10)	4.32 (0.16)
I 8	1.51 (0.05)	0.29 (0.02)	2.77 (0.08)	4.02 (0.14)
**Breast symptoms**				
I 20	2.78 (0.09)	−0.12 (0.02)	1.86 (0.04)	2.75 (0.06)
I 21	3.38 (0.13)	0.37 (0.02)	1.95 (0.04)	2.73 (0.06)
I 22	2.80 (0.09)	0.23 (0.02)	2.01 (0.04)	2.93 (0.07)
I 23	1.71 (0.05)	0.17 (0.02)	2.26 (0.05)	3.28 (0.09)
**Arm symptoms**				
I 17	2.78 (0.10)	−0.46 (0.02)	1.40 (0.03)	2.28 (0.05)
I 18	2.53 (0.08)	−0.10 (0.02)	1.51 (0.03)	2.27 (0.05)
I 19	2.26 (0.07)	0.12 (0.02)	1.82 (0.04)	2.68 (0.06)

a: discrimination b1: difficulty parameter (interviewer endorsing 1 versus ≥2); b2: difficulty parameter (interviewer endorsing 2 versus ≥3); b3: difficulty parameter (interviewer endorsing 3 versus 4).

### Preliminary validation of short form

Table 4 displayed the results for the shortened for each of the four domains. We divided the survivors into four groups according to the quartile of the scores of the short form and the original, respectively. The proportion of respondents correctly predicted was high and similar, as compared to the original scale. The mean difference between the shortened form and the original observed BRST scores and BRBS scores are less than 1; BRBI scores and BRAS scores were less than 2.5. Both the correlation and the weighted kappa were high.

**Table 4:** Prediction of the scores on the original scales. Results for the shortened scales performing best for each of the four domains.

Scale	Items in short scale	Correct (%)^a^	Mean diff. (SD)^b^	Correlation^c^	Kappa^c^
BRBI					
Training set	10,11,12	87.10	1.74 (5.40)	0.982	0.90
Testing set		86.44	1.94 (5.36)	0.983	0.89
BRST					
Training set	2,3,6,8	70.38	0.77 (5.49)	0.921	0.74
Testing set		69.76	0.80 (5.69)	0.916	0.73
BRBS					
Training set	20,21,22	100.00	0.15 (4.79)	0.963	1.00
Testing set		100.00	0.18 (5.01)	0.960	1.00
BRAS					
Training set	17,18	100.00	−2.04 (6.79)	0.951	1.00
Testing set		100.00	−2.17 (6.97)	0.951	1.00

^a^Percent correctly predicted scale scores; ^b^Mean difference between predicted and observed scale scores; ^c^Correlation and weighted kappa between predicted and observed scores.

## Discussion

The expansion of study on the cancer survivors’ quality of life, and the great need for well-validated questionnaires suitable for evaluating the construct with more than a single dimension, led us to conduct this study to develop a shortened version of the EORTC QLQ-BR23. One of the important assumptions of IRT analysis is unidimensionality^24^, referring to the question whether the items measure the same potential traits. All the items in the data evidently measured some aspects of quality of life, therefore, we analyzed each dimension separately. Since the sexual function only have two items, and during the past four weeks more than 80% participants reported they had no interested in sex and had no sexually active, less than 2% participants reported the response of “Quite a bit” or “Very Much”. This phenomenon might be attributed to the fact that the women were shy and reserved when talked about sexuality, especially old women in China. Therefore, our study here is on the BRBI, BRST, BRBS and BRAS scales.

Generally, when the standard error is less than 0.2 we consider the item has a high quality; while the standard error is less than 0.25 we define the item as acceptable but needs to be improved; whereas, when the standard error is more than 0.25, we define the item as poor quality and consider deleting it^25^. According to the formula: I = 1/σ^2^ ^26^, the total item information should be higher than 16. The EORTC QLQ-BR23 consists of 23 items, therefore, the information of each item greater than 0.70 (16/23) was defined as good quality, and if the information of each item more than 1.09 (25/23) then defined as excellent. For the dimension of body image, breast symptoms and arm symptoms, item 9, 23 and 19 were deleted based on the information criterion. However, this also reminded us that this dimension might need to be improved when used in Chinese population.

The evaluations for the information of systemic therapy side effects scale were all less than 0.70. In order to maintain the balance of the content dimension of the whole scale, we kept the four items with the highest information in this dimension. The evaluations of the systemic therapy side effects showed a slightly poorer agreement than for the other three scales. Some of the reasons might due to the source of our sample that mainly involved with the member of the Cancer Recovery Clubs and long-term survivors. Most of the participants were in an early stage of the disease (TNM classification 0 or 1 or 2), and had a high quality of life. Therefore, they had fewer symptoms of systemic therapy side effects now, resulting in less information on the systemic therapy side effects to the EORTC QLQ-BR23 in our study.

The BRBI scores predicted with item 10, 11 and 12, the BRBS scores predicted with item 20, 21 and 22, and the BRAS scores predicted with item 17 and 18, were all in a great agreement with the original scales. The correlation and weighted kappa coefficient of BRBI between predicted and original scores were 0.98 and 0.9, respectively. Using item 10, 11 and 12 may be expected to result in the same findings and conclusions as using the full BRBI scale. The shortened BRBS and BRAS scales were extremely perfect in predicting the original scale scores, the percent correctly predicted scale scores were all 100% and the correlation coefficient were all higher than 0.95.

Unlike classical test theory, results from IRT calibration contain detailed item-level information that can be considered from many useful perspectives^27^. For example, the test characteristic curves and the summation of Item characteristic curves for the entire instrument are especially useful in defining the cutoff value between the shortened and the raw data score, and also useful in estimating of the original scale score. IRT had been used by many other researchers to create short versions of existing instruments^14,28–31^. Some of the previous studies use a similar strategy as reported here for shortening the EORTC QLQ-C30 scale^32–34^. Our results were so consistent with these IRT based prediction methods that it seemed possible to shorten scales and simultaneously provide high precision in predicting the scores on the original scale.

Based on the present study we expect an application of a four-item BRST scale composed by items 2, 3, 6 and 8; a three-item BRBI scale composed by items 10, 11 and 12; a three-item BRBS scale composed by 20, 21 and 22; and a two-item BRAS scale composed by 17 and 18 in a shortened version of the EORTC QLQ-BR23 for breast cancer survivors with severity. In all six items were deleted using the IRT based approach. We hope that some of the single items or scales (i.e., sexual function) will be deleted, and the questionnaire could be cut off by a half, so that it could dramatically expand the scope of application of the questionnaire in the future studies.

A limitation of the study is that the sample was recruited from the Cancer Recovery Clubs, with a long-term survival and a higher quality of life. Therefore, further studies are needed to investigate the results in newly diagnosed breast cancer patients with poorer quality of life. Notwithstanding its limitations, some strengths of our study are still far from being neglected. For instance, the large size of the sample enhanced power of the estimation procedures, and the application of IRT methodologies for identifying a subset of items maximized reliability and maintained adequate precision.

## Conclusions

IRT is an effective analysis method to shorten the scales and simultaneously provide high quality in predicting the scores on the full scale. Prospective validation on newly diagnosed breast cancer patients and with poor QOL is needed for further studies. Given the favorable results for the BRBI, BRST, BRBS and BRAS scales we expect that the shortened version of the EORTC QLQ-BR23 is of potentially practical value for researchers and clinicians.

## Electronic supplementary material

Supplementary information23 Item ICC
