# Supplementary material for: Use of item response theory to develop a shortened version of the EORTC QLQ-BR23 scales
Source: Sci Rep. 2019 Feb 11;9:1764. doi: 10.1038/s41598-018-37965-x (PMC6370820; doi:10.1038/s41598-018-37965-x)

**Title Page**

**Title:** Use of item response theory to develop a shortened version of the EORTC QLQ-BR23 scales

**Author list:**

**Juan Xia^1^, Zheng Tang^1^, Peng Wu^1^, Jiwei Wang*^1^, Jinming Yu*^1^**

1 Institute of Clinical Epidemiology, Key Laboratory of Public Health Safety, Ministry of Education, School of Public Health, Fudan University, China

*** Corresponding Author:**

Jinming Yu, Institute of Clinical Epidemiology, Key Laboratory of Public Health Safety, Ministry of Education, School of Public Health, Fudan University, 130 Dong-An Road, Shanghai, China, 200032. Tel: (021)54237868; Fax: (021)54237868; E-mail: [jmy@fudan.edu.cn](mailto:jmy@fudan.edu.cn).

Jiwei Wang, Institute of Clinical Epidemiology, Key Laboratory of Public Health Safety, Ministry of Education, School of Public Health, Fudan University, 130 Dong-An Road, Shanghai, China, 200032. Tel: (021)54237898; Fax: (021)54237898; E-mail: [jiweiwang@fudan.edu.cn](mailto:jiweiwang@fudan.edu.cn).


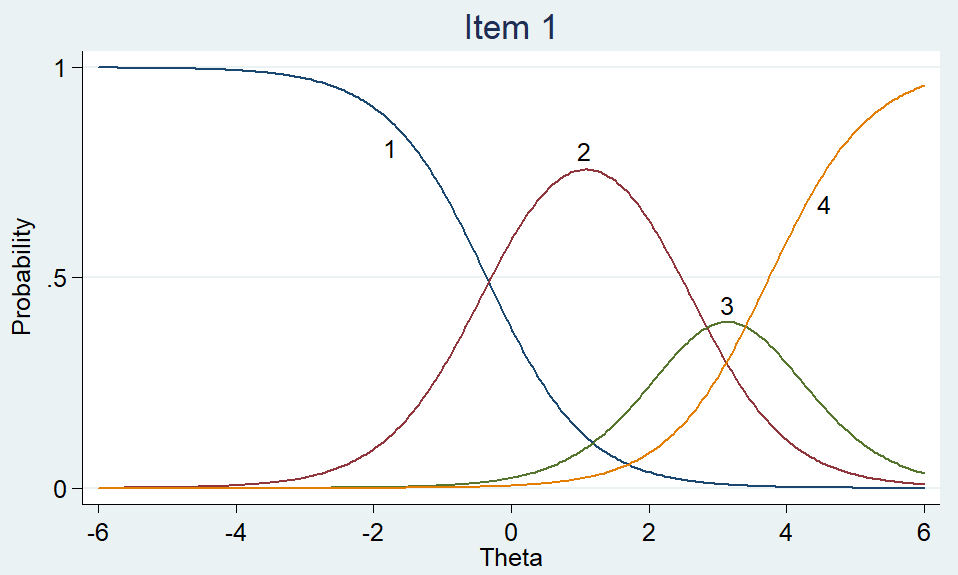


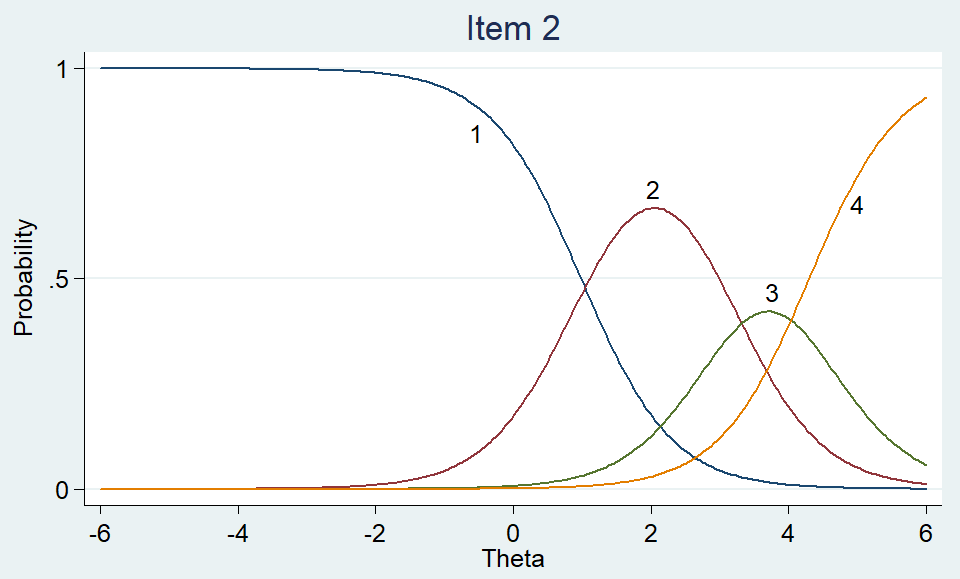


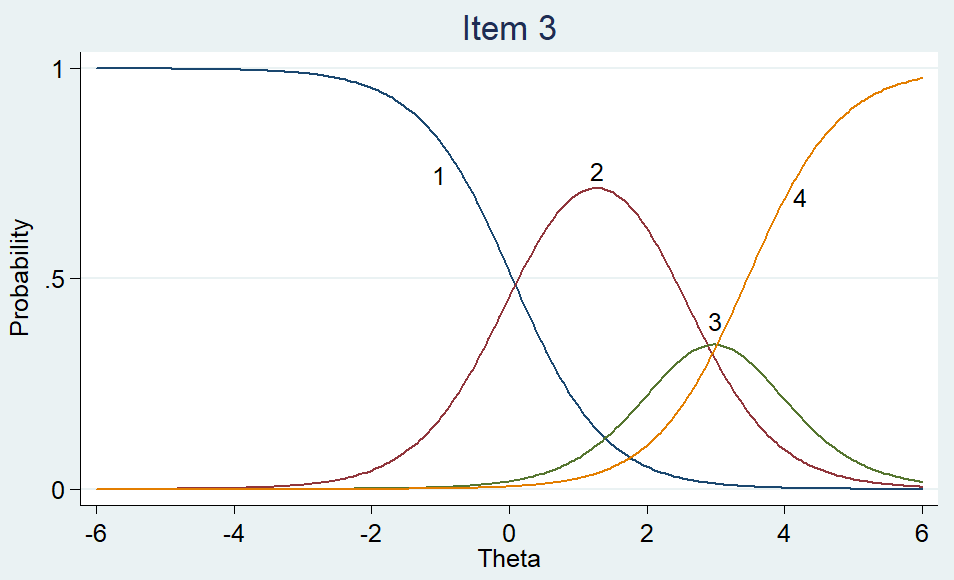


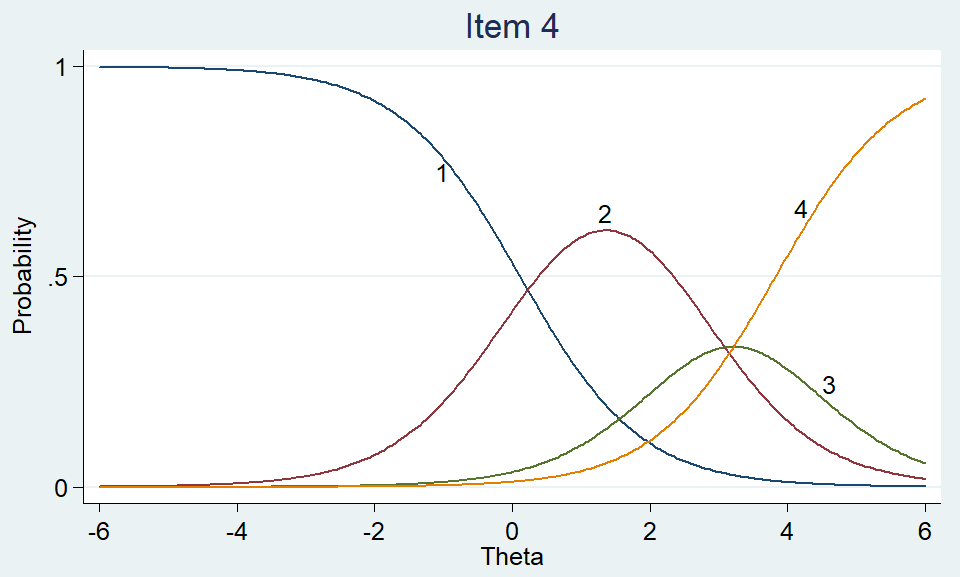


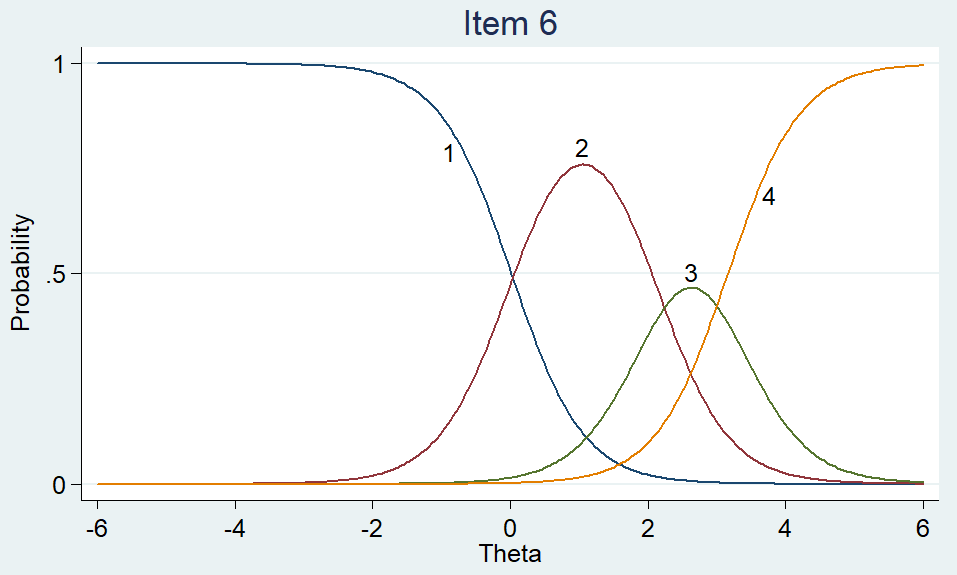


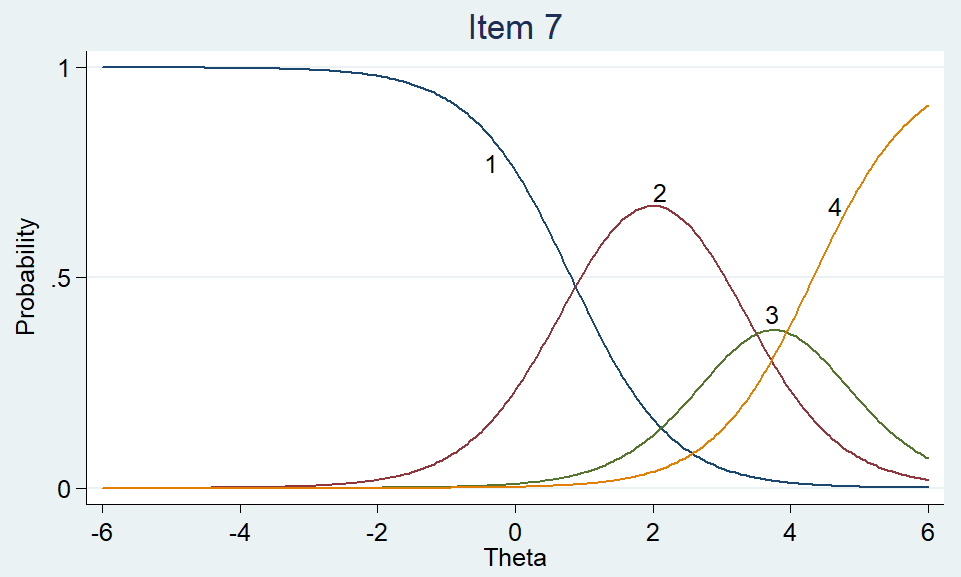


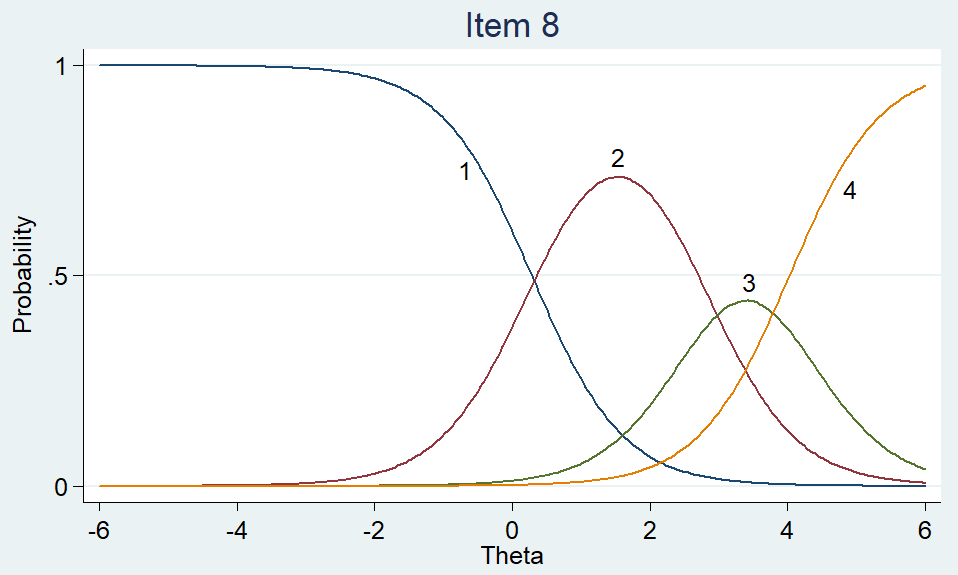


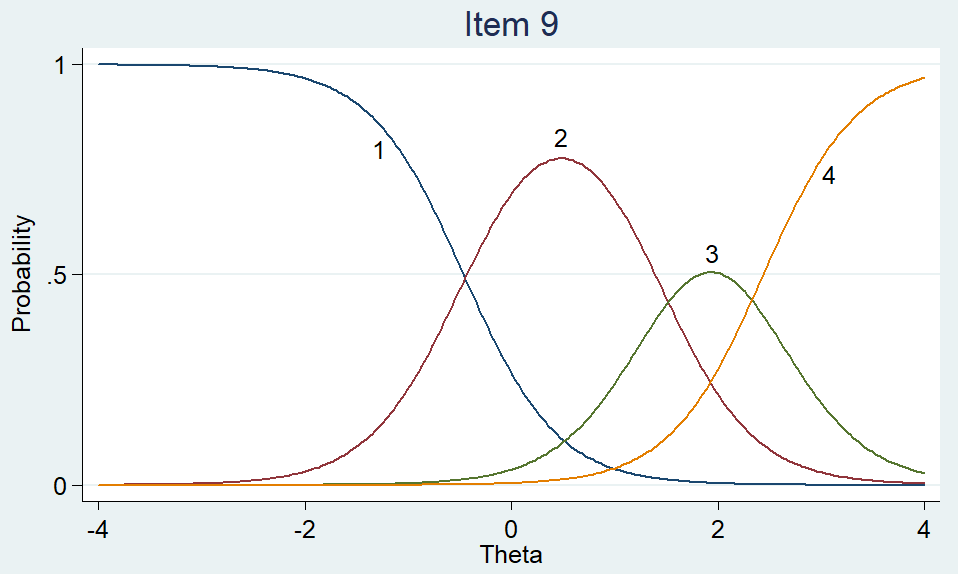


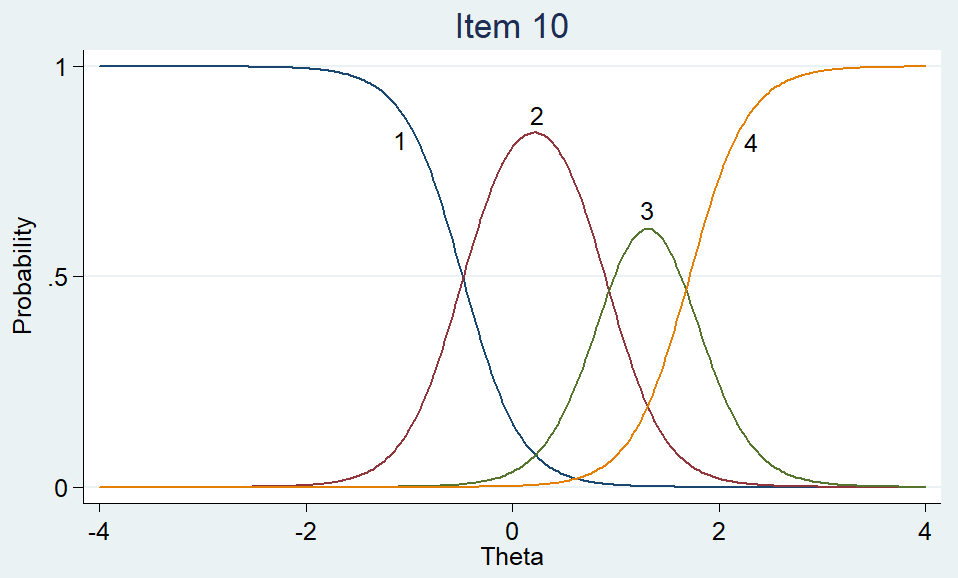


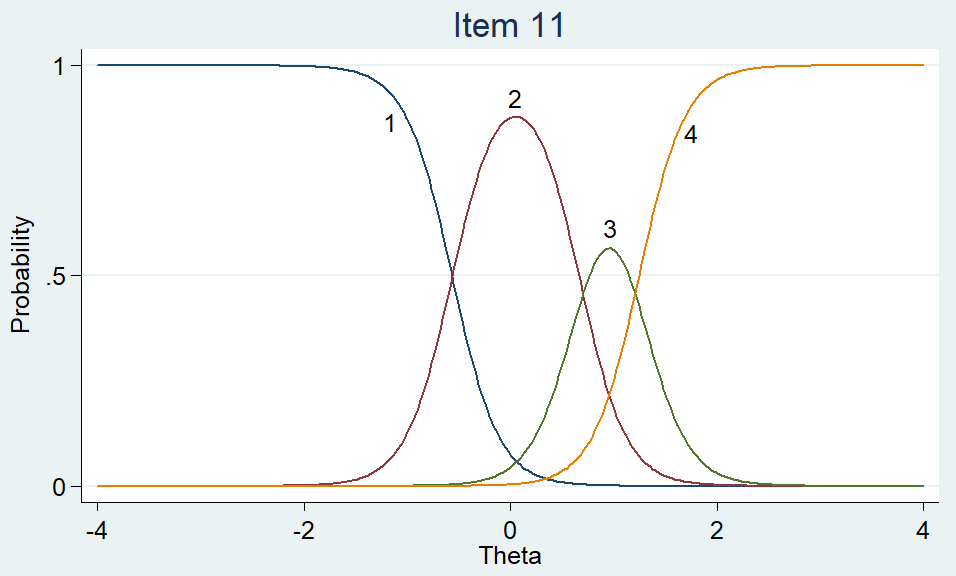


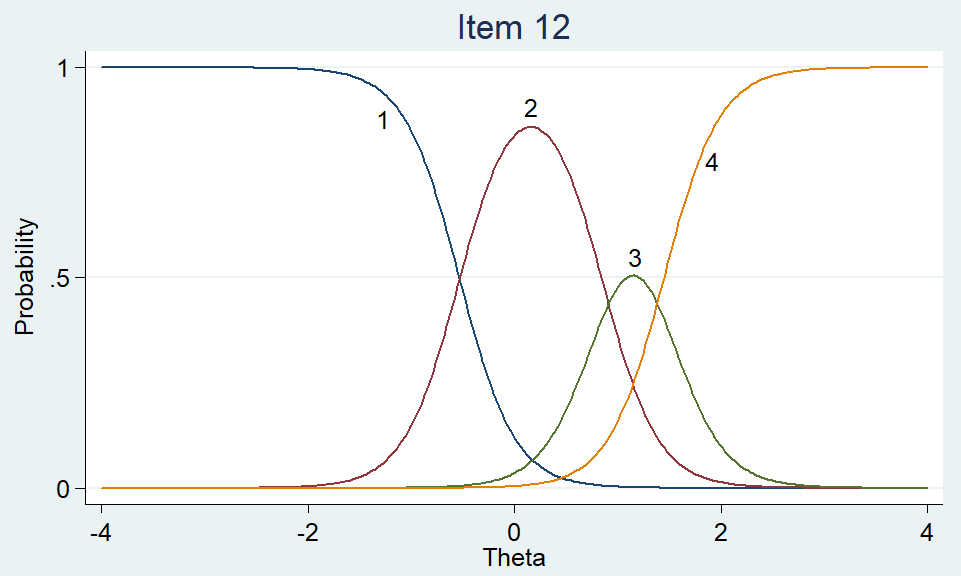


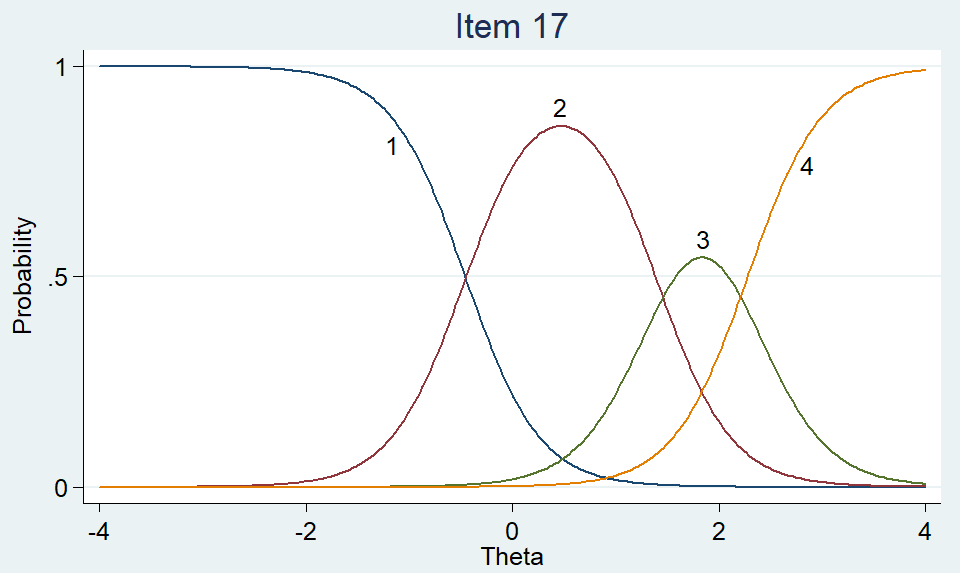


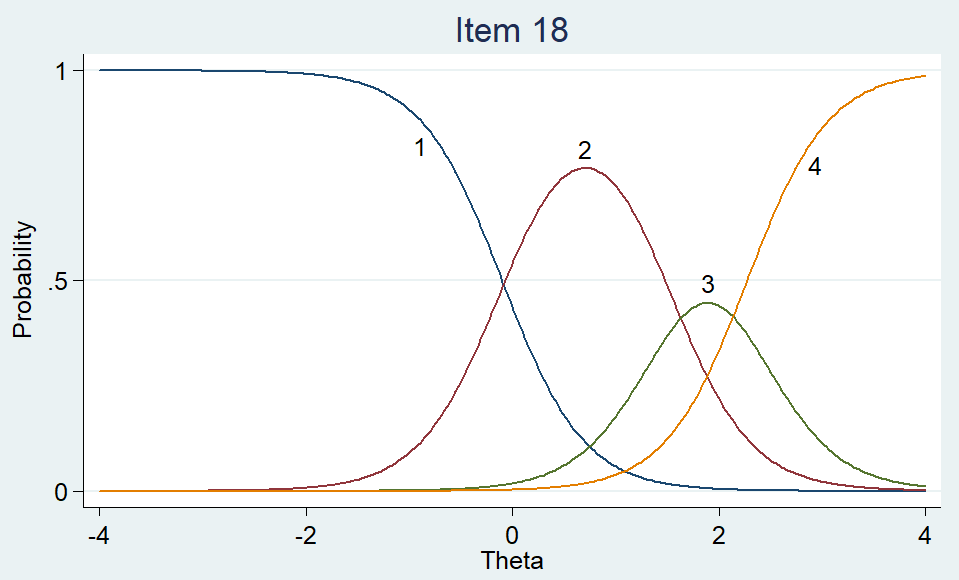


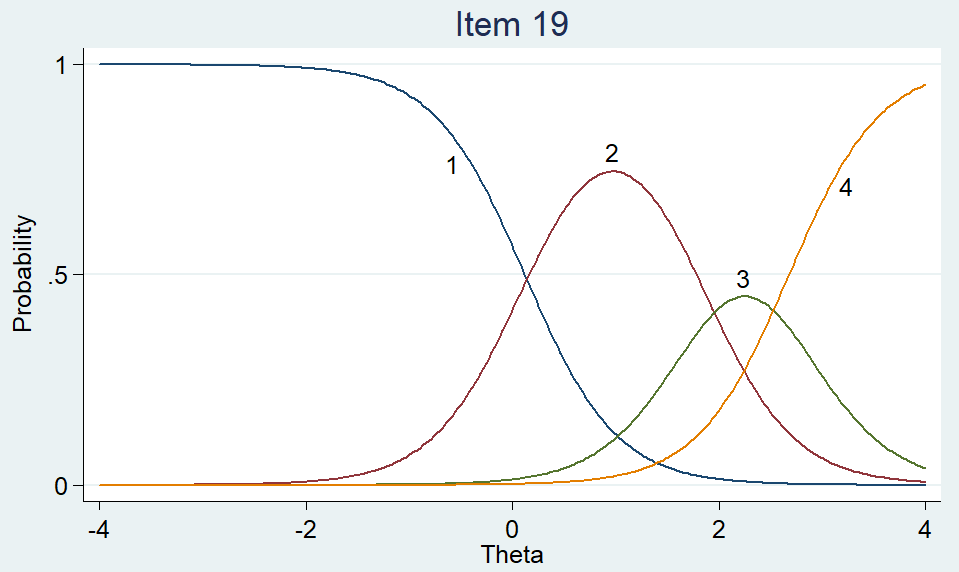


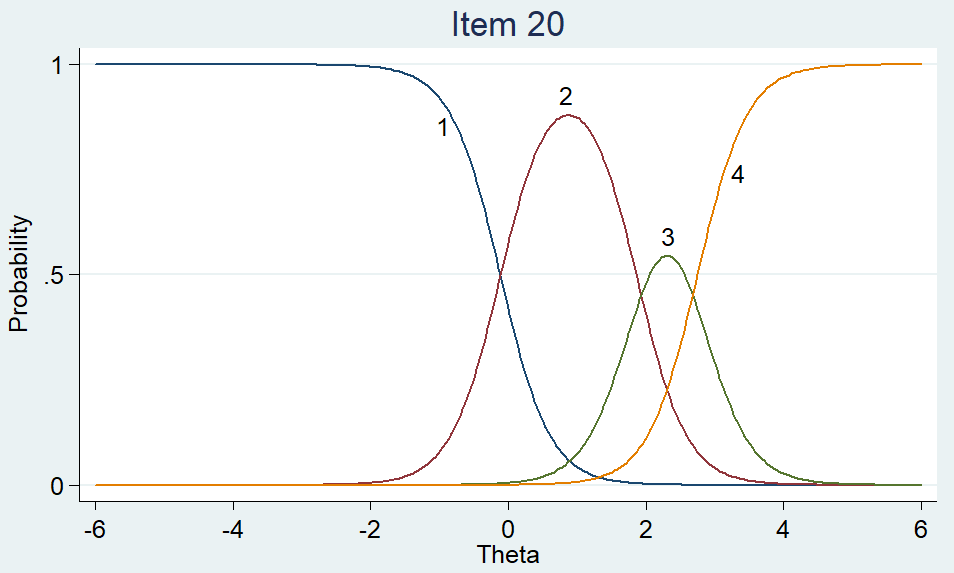


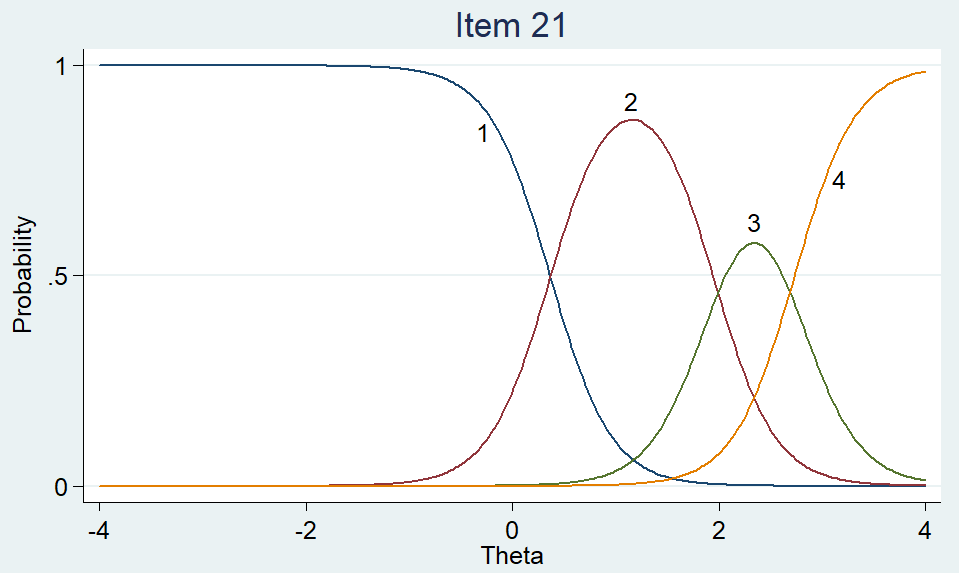


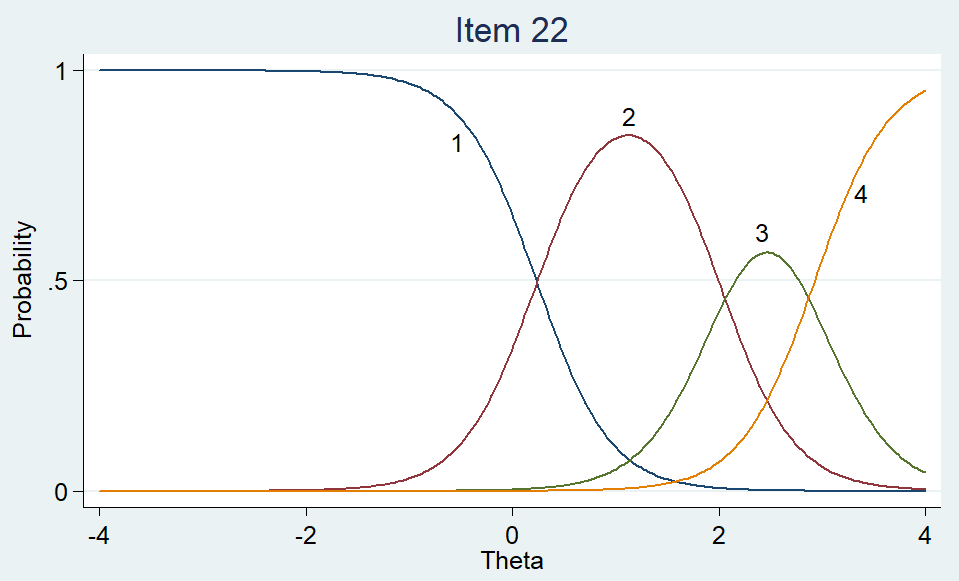


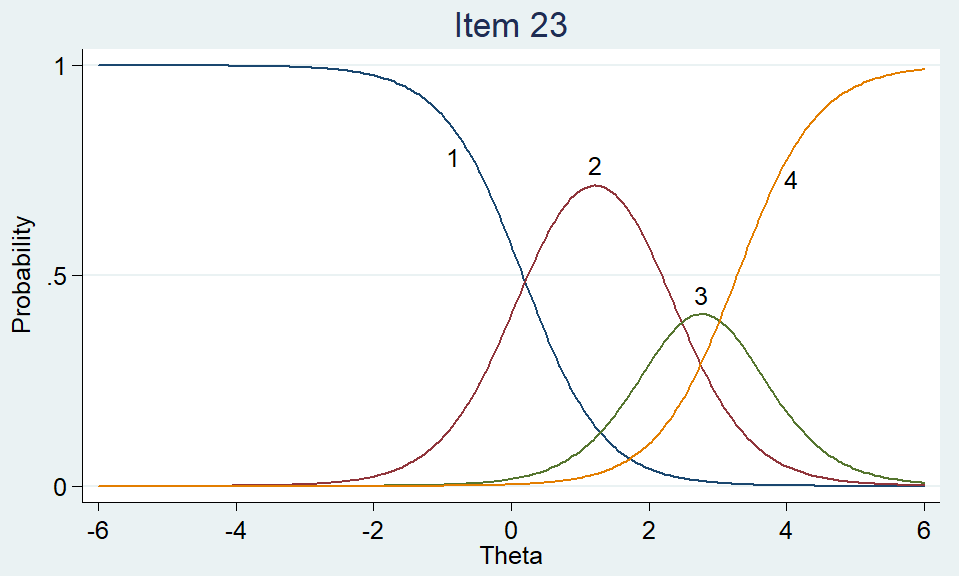

Supplement: Supplementary file 1 — Supplementary information23 Item ICC [file 41598_2018_37965_MOESM1_ESM.docx]
